# Single center experience on efficacy and safety of Aprepitant for preventing chemotherapy-induced nausea and vomiting (CINV) in pediatric Hodgkin Lymphoma

**DOI:** 10.1371/journal.pone.0215295

**Published:** 2019-04-12

**Authors:** Giovanna Giagnuolo, Salvatore Buffardi, Francesca Rossi, Fara Petruzziello, Chiara Tortora, Isabella Buffardi, Nicoletta Marra, Giuliana Beneduce, Giuseppe Menna, Rosanna Parasole

**Affiliations:** 1 Department of Pediatric Hemato-Oncology, Santobono-Pausilipon Hospital, Naples, Italy; 2 Dipartimento della donna, del bambino e di chirurgia generale e specialistica, Università della Campania Luigi Vanvitelli, Naples, Italy; University of Texas M. D. Anderson Cancer Center, UNITED STATES

## Abstract

Chemotherapy-induced nausea and vomiting (CINV) is a distressing treatment side-effect that could negatively affect children’s quality of life (QoL). Different scoring systems for CINV were applied and different antiemetic drugs were used; however, few studies have been performed in children undergoing chemotherapy with Aprepitant. Herein, we report a pediatric experience on efficacy and safety of Aprepitant as part of triple antiemetic prophylaxis, in a cohort of thirty-two children and adolescents with Hodgkin Lymphoma (HL), treated with moderate/highly emetogenic chemotherapy (MEC/HEC) regimens in a single Hemato-Oncology Institution. The triple therapy was compared to standard antiemetic therapy in a cohort of twenty-three HL patients (control group). Aprepitant therapy was associated to a significant decrease of chemotherapy-induced vomiting (*p* = 0.0001), while no impact on the reduction of nausea was observed; these observations were also confirmed by multivariate analysis (*p* = 0.0040). Aprepitant was well tolerated and the most commonly reported adverse events were neutropenia and hypertransaminasemia. No significant differences on the toxicity were observed between the two compared groups. Our experience on Aprepitant efficacy and safety, associated with feasibility of orally administration, suggests a possible widespread use of the drug to prevent pediatric CINV.

## Introduction

Chemotherapy-induced nausea and vomiting (CINV) is a common and distressing side-effect of cancer therapy; its prevention is needful in order to avoid the negative impact of these symptoms on patients’ quality of life (QoL) [1].

The combination of corticosteroid and serotonin receptor antagonists (5-HT_3_-RAs) has been the gold standard of CINV prevention related to highly (HEC) and moderately emetogenic chemotherapy (MEC) [2–3], until the introduction of Aprepitant [4], a selective neurokinin-1 (NK-1) receptor antagonist, that, with its long lasting, central and peripheral, antiemetic activity, has provided a significant improvement in control of both acute and delayed CINV, with considerable benefits on treatment compliance [5–6]. In 2013, the Pediatric Oncology Group of Ontario (POGO) Guidelines recommended that children scheduled for HEC should be administered antiemetic prophylaxis of ondansetron plus dexamethasone with or without aprepitant; however, this recommendation applied only to children aged ≥12 years [7].

Current MASCC (Multinational Association of Supportive Care in Cancer) guidelines recommend a triple antiemetic prophylaxis consisting of 5HT3-RA, dexamethasone and Aprepitant in pediatric patients, six months or older, receiving HEC [8]. Aprepitant is also recommended, in association with 5HT3-RA, for children receiving MEC who can not have dexamethasone [8]. The efficacy and safety of aprepitant on emesis in pediatric patients has been evaluated in randomized trials that reported a significantly higher CIV control in children treated with HEC who received aprepitant plus 5-HT3-RA with/without dexamethasone compared to those who received only 5-HT3-RA with/without dexamethasone [9–10]. Although, different scoring systems have been proposed to identify potential risk factors for CINV and different antiemetic drugs are using, however, limited studies have been performed in children undergoing chemotherapy [11–12].

This retrospective, monocentric and nonrandomized study evaluated the efficacy and safety of Aprepitant for the prevention of chemotherapy-induced nausea and vomiting in children and adolescents with Hodgkin Lymphoma (HL) comparing a triple-therapy regimen of Aprepitant plus dexamethasone and ondansetron (Aprepitant group) with a standard therapy (Control group).

## Materials and methods

### Study design and patients

The study population was composed of male and female children and adolescents with HL, aged ≥12 years, diagnosed from January 2015 and treated with MEC/HEC regimens [13], such as COPP/ABV, ABVD, IEP/OPPA, OEPA, COPDAC-28, DECOPDAC-21 or Bendamustine [7,14]; Table 1 shows chemotherapy regimens, dosing frequency, route of administration and potential emetogenicity. All relevant clinical data were collected from the patient’s electronic medical records.

**Table 1:** Chemotherapy regimen and acute emetogenic potential.

Regimen	Schedule	Potential Emetogenicity
**COPP/ABV**	Cyclophosphamide: 600 mg/m^2^ i.v. day 1 Vincristine: 1.4 mg/m^2^ i.v. day 1 Prednisone: 40 mg/m^2^ p.o. days 1 to 14 Procarbazine: 100 mg/m^2^ p.o. days 1 to 7 Doxorubicin: 35 mg/m^2^ i.v. day 8 Bleomycin: 10 mg/m^2^ i.v. day 8 Vinblastine: 6 mg/m^2^ i.v. day 8	High level
**ABVD**	Doxorubicin: 25 mg/m ^2^ i.v. days 1, 15 Bleomycin: 10 mg/m^2^ i.v. days 1, 15 Vinblastine: 6 mg/m^2^ i.v. days 1, 15 Dacarbazine: 375 mg/m^2^ i.v. days 1, 15	High level
**IEP**	Ifosfamide: 2000 mg/m^2^ i.v. days 1 to 5 Etoposide: 120 mg/m^2^ i.v. days 1 to 5 Prednisone: 100 mg/m^2^ p.o. days 1 to 5	Moderate level
**OPPA**	Vincristine: 1.5 mg/m^2^ i.v. days 1 & 14 Procarbazine: 100 mg/m^2^ p.o. days 0 to 13 Prednisone: 60 mg/m^2^ i.v. days 0 to 13 Doxorubicin: 35 mg/m^2^ i.v. days 0, 14	High level
**OEPA**	Prednisone/Prednisolone: 60 mg/m^2^/day p.o. days 1–15 Vincristine: 1.5 mg/m^2^ i.v., days 1 + 8 + 15 Doxorubicin: 40 mg/m^2^ i.v days 1 + 15 Etoposide: 125 mg/m^2^ i.v. days 1–5	Moderate level
**COPDAC-28**	Prednisone/Prednisolone: 40 mg/m2/day p.o. days 1–15 Dacarbazine: 250 mg/m^2^ i.v. days 1–3 Vincristine: 1.5 mg/m^2^ i.v. days 1 + 8 Cyclophosphamide: 500 mg/m^2^ days 1 + 8	High level
**DECOPDAC-21**	Prednisone/Prednisolone: 40 mg/m^2^/day p.o. days 1–8 Dacarbazine: 250 mg/m^2^ i.v. days 1–3 Vincristine: 1.5 mg/m^2^ i.v. days 1 + 8 Cyclophosphamide: 625 mg/m^2^ i.v. days 1–2 Etoposide: 100 mg/m^2^/day i.v. days 1–3 Doxorubicin:25 mg/m^2^ i.v. day 1	High level
**Bendamustina**	Bendamustina: 90 mg/m^2^ i.v. days 1–2	Moderate level

For the Aprepitant group (AP group), thirty-two patients (18 females/14 males; mean age 14.3 years) with HL (30 nodular sclerosis, 1 lymphocyte predominant variety and 1 lymphocyte depletion type), at different stage of disease, were enrolled until March 2018. Eighteen chemotherapy-naïve patients received first line therapy with COPP/ABV or ABVD regimen according to the HL-04 protocol of the Italian Hematology and Oncology Association (AIEOP), ten patients received first line chemotherapy with OEPA plus COPDAC-28 or DECOPDAC-21, according to the EURONET-PHL-C2 protocol (NCT02684708). Three refractory patients had second line therapy with IEP/OPPA and the latter received fourth line treatment with Bendamustine for relapse after autologous bone marrow transplantation. A total of 166 cycles were analyzed with an average of 5.1 cycles per person (range 3–11). Baseline characteristics of patients are reported in Table 2. According to the Supportive guidelines for children receiving HEC/MEC regimens [7–8], this group of patientsreceived Aprepitant as part of triple antiemetic prophylaxis during each cycle of chemotherapy, all patients received adult-like Aprepitant dose (125 mg given orally on Day 1 and 80 mg on Days 2 and 3) in association with i.v. ondansetron (4 mg/mq) and i.v. dexamethasone (0.5–2 mg/kg) before each chemotherapy cycle or oral prednisone (40–60 mg/mq in three daily administrations for 14–15 days), as part of HL protocol; no steroids dose adjustment was applied and no Aprepitant dosing was converted to mq/kg, as recommended by recent guidelines [14]. Written informed consent was obtained from patients’ parents, and assent from children aged > 14 years, before the first Aprepitant administration.

**Table 2:** Patients baseline characteristics by treatment group.

	Aprepitant	Control	p-Value
**Patients, n**	32	23	
**Males, n (%)**	14 (43,8)	12 (52,2)	0.59
**Females, (%)**	18 (56,2)	11 (47,8)	
**Age, years (mean±SD)**	14.3±1.79	11.6±2.74	<0.0001

In our institute, all patients during intensive chemotherapy, complete a questionnaire in order to identify the occurrence of side effects. such as nausea, vomiting, fatigue, discomforts or pain. This questionnaire included “Baxter Retching Faces” (BARF) nausea scale [15] to assess the intensity of nausea, and the number of vomiting episodes. Supplementary administrations of antiemetic medications for uncontrolled CINV were reported by the physicians or nurses in the clinical records.

The control group (CTR group) consists of twenty-three patients (11 females/12 males; mean age 11.6 years) with HL (20 nodular sclerosis, 2 lymphocyte predominant variety and 1 with a mixed cellularity type), at different stage of disease. Eighteen patients of the this group received the diagnosis before 2015, while 5 patients received the diagnosis contemporary to the patients of AP group. Twenty patients received first line therapy with COPP/ABV or ABVD regimen according to the HL-04 protocol of AIEOP, in detail, eight of these patients received ABVD therapy and twelve received COPP/ABV. One patient received OEPA/COPDAC treatment according to the international protocol EuroNet-PHL-C2, another one received a second line therapy with IEP/OPPA and the last one received DHAP therapy [16] for a late relapse.

This group of patients was treated with a standard anti-emetic therapy which includes i.v. ondansetron (4 mg/mq) and i.v. dexamethasone (0.5–2 mg/kg) before each chemotherapy cycle. Considering their young age (<12 years), the poor therapy compliance and that, at the time of enrollment, some of these, had already started a standard prophylaxis, also the patients who received the diagnosis contemporary to the AP patients, received the standard anti-emetic protocol.

Baseline characteristics of patients are reported in Table 2.

The study was approved by the “Cardarelli-Santobono-Pausilipon” Ethics Committee, in accordance with the Declaration of Helsinki.

### Statistical analysis

All the analyses were conducted by using Statgraphics CENTURION XV.II (Adalta, Arezzo, Italy; STATPOINT TECHNOLOGIES INC., Virginia, USA). Categorical data were expressed as percentage and continuous data as mean±SD.

The Fisher's exact test or the chi-square test was used to evaluate the difference among categorical variables distribution.

Multivariate analysis, using a general linear model, was performed to explore the effect of independent variables significantly associated with emesis in the univariate analysis. A *p* ≤ 0.05 was considered statistically significant.

## Results

### Efficacy

The efficacy of antiemetic prophylaxis was evaluated through a questionnaire given to the patients or their relatives after each cycle. The questionnaire assessed the incidence of nausea and vomiting during chemotherapy, starting with the administration of the first chemotherapy dose of the block and continuing until 24 hrs after administration of the last dose and in the subsequent 96 hours (delayed CINV) after cycles. Patients were asked to provide a global evaluation of their nausea intensity using a scale from 1 to 10 accordingly to the “Baxter Retching Faces (BARF)” scale [15], the number of vomiting episodes and the occurrence of other adverse events. Supplementary administrations of antiemetic medications for uncontrolled CINV were reported into the clinical records.

Complete response was defined as absence of nausea and emesis during cycle and until the first 24 hours (acute CINV) after last dose of chemotherapy. Response was considered partial when patients showed nausea in absence of vomiting episodes. Finally, patients who failed to control vomiting, without rescue antiemetic medication, were considered non-responders [13]. Delayed CINV was defined as nausea and vomiting >24–96 hrs after cycle discontinuation.

**Fig 1:**
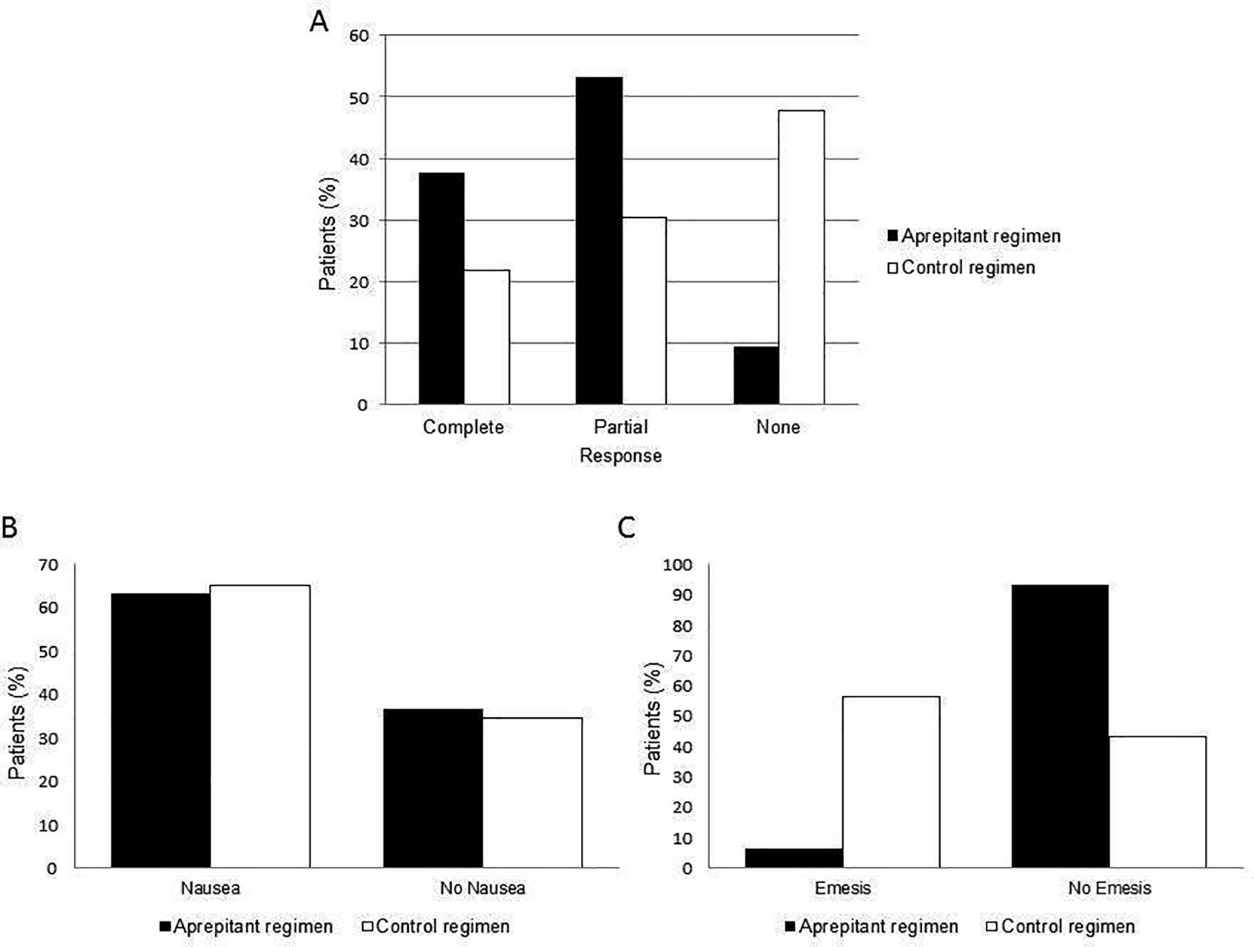
Efficacy. A) Percentage of patients of Aprepitant and Control group with complete, partial or none response. Percentage of patients of Aprepitant and Control group who experienced nausea (B) and emesis (C).

Data analysis showed a significant difference between the AP group and the CTR group (*p =* 0.0026) (Fig 1A).

In AP group twelve patients (37,5%) showed complete absence of acute CINV, 17 (53,1%) reported nausea with a variable intensity from 1 to 8 in a scale from 1 to 10 [15], and only 3 patients (9,4%) experienced few episodes of emesis (1, 2 and 4 episodes, respectively) within 24 hours from starting chemotherapy, during only one cycle (first, third and fourth, respectively). Among patients with partial response, one received only aprepitant plus steroids as antiemetic prophylaxis, because she could not receive ondansetron due to long QT-syndrome. No patients experienced delayed CINV. If we analyze, retrospectively, CINV response according to the current definition of complete CINV control (defined as either no vomiting and nausea or no vomiting) or failure (defined 3 or more emetic episodes in 24 hr) [13], all, except one patient, obtained a complete response (96,8% responders). In CTR group, 5 patients (21,74%) showed complete absence of acute CINV, 7 (30,43%) reported nausea without vomiting and 11 patients (47,82%) experienced vomiting.

Moreover, to give more strength to the obtained result, we analyzed individually the contribution of the nausea and vomiting events (Fig 1B and 1C).

In AP group nausea occurred with a percentage of 63,33% compared to 65,21% observed in the no AP group (*p* = 0.8873). The administration of Aprepitant would seem to have no impact on the reduction of nausea.

Instead, it is surprising to observe how the vomiting occurred only in 6,66% of patients in the AP group compared to 56,52% observed in the CTR group (*p* = 0.0001).

The administration of Aprepitant increased significantly the efficacy of the standard antiemetic therapy reducing the episodes of emesis.

#### Multivariate logistic regression analysis

To confirm the relation between emesis and AP therapy, a multivariate logistic regression analysis was performed (Table 3), using emesis as a dependent variable, Aprepitant therapy and other potential confounding factors like age, sex, ABVD and COPP/ABV therapy as conditioning variables. The analysis showed that emesis was still significantly associated with Aprepitant therapy (*p* = 0.0040) as well as in the univariate analysis. Moreover also ABVD therapy was found to be associated with emesis (*p* = 0.0369) suggesting it as highly emetogenic.

**Table 3:** Logistic regression.

**Analysis of Variance for emesis**					
**Source**	**Sum of Squares**	**df**	**Mean Square**	**F-Ratio**	**p-Value**
Model	4.18991	5	0.837981	6.00	**0.0002**
Residual	6.56481	47	0.139677		
Total (Corr.)	10.7547	52			
**Single factor contribution**					
**Source**	**Sum of Squares**	**df**	**Mean Square**	**F-Ratio**	**p-Value**
EMEND	1.27609	1	1.27609	9.14	**0.0040**
Sex	0.00552304	1	0.00552304	0.04	0.8432
Age	0.0105296	1	0.0105296	0.08	0.7849
ABVD	0.644297	1	0.644297	4.61	**0.0369**
COPP/ABV	0.000404204	1	0.000404204	0.00	0.9573

### Safety

To evaluate the safety of Aprepitant, we analyzed chemotherapy-related adverse events in order to exclude any negative contribution of Aprepitant.

Hematological and non-hematological toxicity was evaluated according to CTCAE criteria (v 4.02: Sept. 15, 2009) [17] at the end of each chemotherapy cycle in both groups.

**Fig 2:**
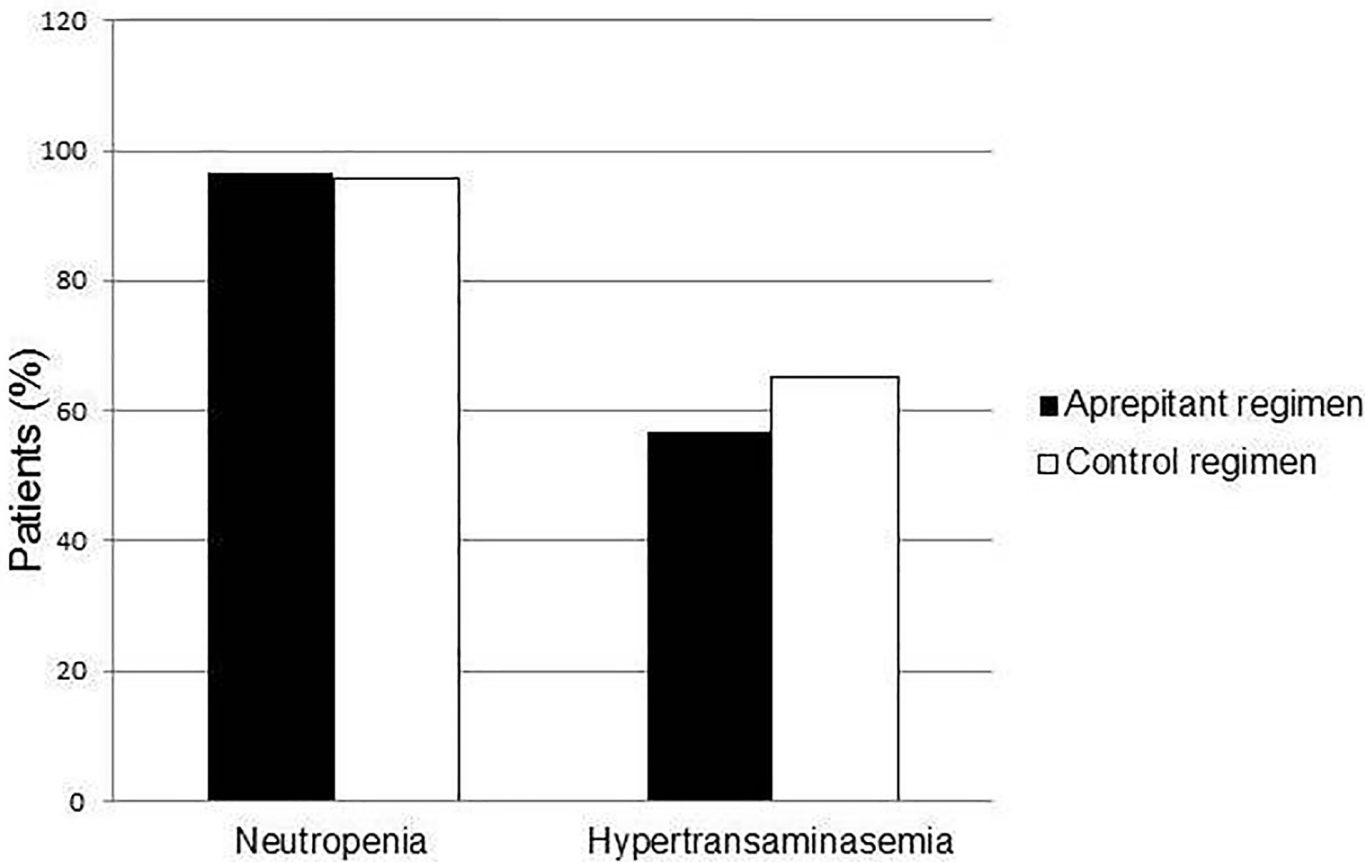
Safety. Percentage of patients of Aprepitant and Control group with neutropenia or hypertransaminasemia.

The most commonly reported adverse events were neutropenia and hypertransaminasemia. No significant differences can be observed between the two compared groups (*p* = 0.37 and *p* = 0.52) (Fig 2).

These events were related to chemotherapy treatment rather than to the administration of Aprepitant.

Hospitalization, due to a severe side effect, was necessary in 3 patients in the AP group: 2 patients with pneumonia who received endovenous antibiotic therapy and another one with Clostridium difficile infection. All the episodes occurred during grade IV neutropenia and they completely resolved at the hematology recovery, without sequelae. These adverse events were expected and neutropenia-related, induced by chemotherapy and not avoidable, according to Liverpool ADR causality [18]. Moreover, a grade III anemia, requesting transfusion, was reported in 3/32 patients of AP group (9,4%) and in 1/23 patients of CTR group (4,5%); instead, a grade IV thrombocytopenia, needing platelets transfusion, was observed only in one patient of AP group, heavily pretreated. Additional side effects were hyperglycemia, diarrhea, mucositis, hyponatremia. No grade III-IV CINV was observed.

#### Correlation between emesis and chemotherapy

In a first general analysis, we also evaluated the frequency of emesis episodes in relation to the different chemotherapy regimen administered to enrolled patients. The statistical analysis showed a strong correlation between emesis and ABVD therapeutic regimen, considering that 77,78% of the patients experienced emesis events (*p* = 0.0003). The result is certainly to be confirmed since the group of patients who received ABVD regimen is too small. Instead, it has not been demonstrated a statistically significant correlation (*p* = 0.1763) with the COPP/ABV regimen; only 20,69% of patients who received this regimen reported emesis episodes.

Moreover, we evaluated the occurrence of emesis in relation to the total number of chemotherapy cycles administered. Statistical analysis showed that 60% of patients experienced vomiting received three cycles of chemotherapy, 33% six cycles and 6,67% five cycles. In contrast, in patients who did not have vomiting, 42% received four total cycles and 47,37% six cycles. The higher incidence of emesis occurs in patients who received three total cycles (*p* = 0.0001) suggesting that it can be correlated to toxic accumulation of chemotherapeutic drugs.

## Discussion

Nausea and vomiting (CINV) are common side effects of cancer therapy that can cause significant negative impacts on patients’ QoL and on their ability to comply with therapy [1]. Despite advances in the prevention of chemotherapy-induced CINV, these side effects remain among the most distressing for patients.

The combination of corticosteroid and serotonin receptor antagonists (5-HT_3_-RAs) represents the gold standard of CINV prevention related to highly (HEC) and moderately emetogenic chemotherapy (MEC).

Aprepitant, the first substance P/neurokinin 1 (NK_1_) receptor antagonist to be approved by the FDA, is now available for oral use with corticosteroids and selective serotonin (5-HT_3_) receptor antagonists to prevent CINV caused by highly emetogenic anticancer drugs [4–6].

This retrospective, monocentric and nonrandomized study evaluated the efficacy and safety of Aprepitant for the prevention of chemotherapy-induced nausea and vomiting in children and adolescents with HL, comparing a triple-therapy regimen of Aprepitant plus dexamethasone and ondansetron with a standard therapy.

Our experience suggests that Aprepitant is effective and safe in pediatric and adolescent affected by HL, who underwent MEC/HEC. Aprepitant improves the performance of the standard antiemetic therapy, reducing significantly the chemotherapy-induced vomiting (*p* = 0.0001).

However, according to literature, no significant differences were observed between the groups for nausea control; NK-1 receptor inhibitors seem to be highly effective reducing the occurrence of vomiting but with less impact on the risk for any emetic symptoms like nausea [19].

Moreover, no delayed CINV were reported in patients of Aprepitant group and no unexpected toxicities were observed. When the treatment groups were compared for drug-related clinical adverse events, no significant differences were observed.

We hypothesize that the most common side effects such as neutropenia and anemia and even the two infective episodes were related to chemotherapy-induced myelotoxicity rather that to Aprepitant administration, despite major incidence of Aprepitant-related febrile neutropenia was reported [20]. Overall the events were considered not avoidable despite the application of prevention strategies.

In conclusion, our encouraging results, even if in a limited but homogenous cohort of pediatric patients, prompt us to recommend the use of Aprepitant in combination with steroid and 5-HT3 receptor antagonists in HL treatment, since the reduction of CINV represents a considerable advantage on quality of life and treatment adherence.

Aprepitant efficacy, associated with the feasibility of orally administration and safety, suggests the possibility of its widespread use for preventing CINV in cancer children receiving HEC/MEC.
